# Negative Life Events, Social Ties, and Depressive Symptoms for Older Adults in China

**DOI:** 10.3389/fpubh.2021.774434

**Published:** 2022-01-20

**Authors:** Hangqing Ruan, Ke Shen, Feinian Chen

**Affiliations:** ^1^Department of Sociology, University of Maryland, College Park, MD, United States; ^2^School of Social Development and Public Policy, Fudan University, Shanghai, China

**Keywords:** negative life events, social ties, depressive symptoms, older adults, China

## Abstract

Although it is widely acknowledged that older adults who have gone through negative life events are more likely to develop depression, there is limited evidence on whether and which type of social ties moderate this perceived relationship. Based on 2016 and 2018 waves of Chinese Longitudinal Aging Social Survey (4,466 individuals, 8,932 observations), we apply linear fixed effects models and confirm that negative life events are associated with depressive symptoms for older adults (Coef. = 0.35; 95% CIs 0.11–0.61), and social ties are negatively associated with depression (Coef. = −0.08; 95% CIs −0.10 to −0.07). Our study further suggests that the association between negative life events and depressive symptoms is significantly moderated by friendship ties (Coef. = −0.18, 95% CIs −0.30 to −0.07), rather than family ties (Coef. = −0.03, 95% CIs −0.09 to 0.15). Moreover, the buffering effects of friendship ties are more prominent for the less resilient and less privileged groups, namely male, rural, and less educated older adults. Our findings point to the importance of expanding and strengthening social networks for Chinese older adults in promoting their psychological health.

## Introduction

World Health Organization (WHO) names depression among older adults as a key global public health concern (1), which leads to heightened disease burden, poor life quality, and high risk of suicide (2, 3). Unipolar depression or major depressive disorder, characterized by a persistent feeling of sadness or a lack of interest in outside stimuli, affected about 7% of the older adults worldwide in 2017 (1). In China, the age-standardized prevalence rate of depression saw a modest decline from 1990 to 2017 according to the Global Burden of Disease Study, that was as opposed to the global trend. However, it is noteworthy that the prevalence rate of depression for older adults aged over 55 years showed a clear upward trend over the same period and was much higher than that for younger adults (4), which was quite consistent with the global pattern. Another study based on the nationally representative survey in China revealed that 37.4 and 6.6% of older adults aged 61–75 reported depressive symptoms and severe depression, respectively, and the corresponding rates for those aged 75+ were even higher, at 40 and 9.7% in 2012 (5). Against the backdrop of accelerated aging process in China, depression has placed great strains on China's health care system and old-age support.

At present, the pathogenesis of depression has not been clearly identified, with genetic, socioeconomic, environmental, and behavioral factors potentially acting jointly (6–8). Among these factors, stress is acknowledged as an important risk factor for depression (9, 10). Negative life events, such as death of family members, severe illness, relationship crisis, and financial problems, pose tremendous stresses on older adults. A large amount of existing studies have demonstrated that experiencing negative life events are often associated with elevated risk of depression or increased depression severity for older adults in Western (11–13) and Asian contexts (14–16), and the magnitude of effects could vary by different types of life events and across various groups of population (13, 17). However, less attention has been paid to how to buffer the deteriorating impacts of negative life events (12, 18, 19). Social support, as a key coping resource, has been documented to improve an individual's resilience and adaptability, and thus contribute to maintaining mental health in psychological research (20). Therefore, it is essential to incorporate social support to examine its moderating impact on the relationship between adverse life events and depression for older adults.

Social support is normally obtained from interpersonal relationships, both within and outside family. In the context of China and other East Asian societies with strong family network, older adults traditionally resort to family members for assistance and comfort in case of difficulties (21, 22). However, along with radical social transformation and massive migration, China has seen prominent family changes in recent decades, such as decline in average household size from 3.1 persons in 2010 to 2.6 persons in 2020, and large increase in one-person and one-generation households at old ages (23, 24). In this circumstance, support from outside household gets increasingly important and critical. The relative importance of family support and friend support in moderating the adverse impacts of negative life events needs to be further explored.

In this paper, based on the biennial panel data from 2016 and 2018 waves of Chinese Longitudinal Aging Social Survey (CLASS), a nationally representative elderly survey, we examine the association between negative life events and depressive symptoms for older adults, and more importantly, differentiate the moderating roles of family ties and friendship ties in the perceived association.

## Literature Review

### Negative Life Events and Depression

The stress process model provides a guiding framework for understanding the association between negative life events and depression. Based on stress process model, stressors that appear either in the form of disruptive life events or the more persistent hardships are related both to people's social and economic status and then to their health (25). Moreover, exposure to one stressor, regardless of whether it is an event or more chronic hardship, may lead over time to exposure to other, secondary, stressors, a process named as stress proliferation (26). Stress proliferation can result in people's lives becoming mired in clusters of stressors, some of which may contribute to cumulative adversity (27). Indeed, negative life events, such as family upheavals, illness, and injury, have been the focus of aging studies in the past decades because older adults often experience them at an elevated level and face heightened risk of stress proliferation. As such, negative life events has been considered as one of the most important stressors of depression for older adults (12, 28).

A large body of empirical studies have demonstrated that experiencing negative life events are associated with elevated risk of depression at old ages. For example, studies have repeatedly shown that family bereavement is closely related to depression at later life (22, 29–32). Some other studies have also found a positive relationship between depression and other negative events such as incidence of severe illness (11), relationship crisis and financial/work problem (33), or the total number of negative life events (15, 34, 35). In 2002, a meta-analysis of 25 studies about negative life events and depression in 1980s and 1990s concluded that both the specific types of negative life events and total number of events is related to depression at a modest level (*r* = 0.15) for older adults (12). Another meta-analysis about 13 qualitative studies of depression in older ages also list negative life events as important risk factors of depression for older adults (36).

Similar results have been reported in Chinese context. One cross-sectional study with 385 community-dwelling older Chinse adults aged above 60 living in Singapore found that increased number of negative life events significantly increased older adults' depressive symptoms (16). Another two longitudinal studies in Hongkong about older adults aged 70 or above (260 older adults, 1992–1995) (14) and community-dwelling Chinese elderly aged 65 or above (2,630 older adults, 2001–2003) (17) also indicated that experiencing one or multiple events significantly increased the risk of depression for older adults.

In summary, a large amount of studies have demonstrated that negative life events are important risk factors of depression for older adults both in Western and Chinese context. However, less attention has been paid to whether and which type of social ties could moderate this perceived association.

### The Buffering Effect of Social Ties: Family Ties vs. Friendship Ties

Social support is beneficial for mental health because it leads to “regular positive experience” and “a sense of predictability and stability in one's life situation, and a recognition of self-worth [(37), p. 311].” Moreover, social support has been documented as a key coping resource to buffer stresses from negative life events (20), but less is known about the structure or context of such support. It is essential to highlight the “contextual nature of social relation” in understanding the role of social support [(38), p. 84]. Social convoy model provides a theoretical framework to understand the dynamic contexts of social support for older adults. Based on social convoy model, individuals are “surrounded by supportive others who move with them throughout the life course” [(38), p. 84]. As for this study, social convoy model indicates that older adults are surrounded by social ties with dynamic function and of different types.

In East Asian context, with persistent Confucian culture, adult children have always been seen as the most important source of support for older adults (21, 22). Traditional family demographers have overwhelmingly emphasized the importance of family ties (12, 35) and empirical studies also found that living with an adult child could lower older adults depressive symptoms both in China and other East Asian countries like South Korea (39, 40). Some studies also argued that family ties can promote health only if the family relationship is harmonious (41, 42).

However, in recent years, the traditional multi-generational living arrangement among older adults has been declining in China (43). In this context, more and more studies turned to emphasize the importance of social ties outside the household (e.g., support from relatives or friends) (44). Friendship ties have been shown to be positively associated with older adult's happiness (45, 46), emotional well-being (47), self-esteem (48), and lowered suicide attempt (49). In Mainland China, it was also found that strong friendship ties is associated with lower depressive symptoms for rural older adults living alone (44).

Although existing literature highlights the importance of family and friendship ties, they put more attention to their direct effects on mental health and less to how they moderate the association between stressful life events and depression. The only exception is the a study by Chou and Chi (15), which showed that the family network, friendship networks, and confident relationships could ameliorate the deleterious effect of negative life events based on a cross-sectional sample of 411 older adults aged 60+ in Hongkong. To the best of our knowledge, there is no study in Mainland China yet. Moreover, the study by Chou and Chi (15) did not compare the relative moderating roles of family networks and friendship networks. The differential buffering effects of family ties and friendship ties needs more rigorous examination.

### Subgroup Differences in Social Ties' Buffering Effect

The meaning to and significance of social ties for different groups may be considerably different, and therefore, the buffering effects of social ties could vary by gender and socioeconomic status. For instance, studies suggest that the depressive symptoms among older adult is a little bit higher for women than men (50, 51), but male and female older adults use different coping strategies when facing stressful life events (52). Women are known as kin-keeper in the family (53) and have a wider and stronger social ties than men (54). In copying with negative life events in later life, women are more resilient (12), and on the contrary, men are more vulnerable in many situation.

Rural-urban differences have also been noted in China context (55). Numerous studies have examined the China's rural-urban divide in state policy, social and economic structure, health, and access to resources (43, 56–59). Rural older adults have higher depressive symptoms than those in urban area (55). But at the same time, rural older adults have less access to social security resources, which leads to more reliance on family and friend support in copying with stress (60). For example, one study in China found that the negative effect of disadvantaged living arrangement (e.g., living alone or living only with children) could be largely reduced by family and friendship ties in rural China, but this effect is less salient in urban China (44). The same logic can be applied to the educational difference in China. People with lower education often have higher level of depression (50, 51), but they are eligible to lower level of social security and have less access to coping resources (61), which make the less educated rely more on social ties when experiencing negative life events.

## Research Hypothesis

This paper focuses on the resilience of the older adults in China when experiencing negative life events and examines how social ties (including family ties and friendship ties) buffer the impacts of negative life events. Existing literature has documented that negative life events are important risk factors of depression in the later life (12). Therefore, we expect that experiencing negative life events would be detrimental to older adults' subjective well-being. In addition, we also expect that social ties (both family and friendship ties) are beneficial to older adults' subjective well-being.

*Hypothesis 1: Experiencing negative life events will increase older adults' depressive symptoms*.
*Hypothesis 2: Social ties (including family ties and friendship ties) are negatively associated with older adults' depressive symptoms*.

Based on the convoy model of social relations (38), we expect that social ties could buffer the association between negative life events and older adults' depressive symptoms:

*Hypothesis 3: Older adults with stronger social ties have lower depressive symptoms than those with weaker social ties when experiencing negative life events*.

In terms of the buffering effect of social ties, we will distinguish between the family ties and friendship ties. Previous studies have shown that family ties and friendship ties differ in the structure, quality, and function of relationship (44). But less is known about their relative importance in the contexts of negative life events for older adults in China. We expect that the buffering effect of family ties and friendship ties on the negative life events are different.

*Hypothesis 4a: family ties play stronger roles in moderating the association between negative life events and depression than friendship ties*.
*Hypothesis 4b: friendship ties play stronger roles in moderating the association between negative life events and depression than family ties*.

In addition, we also hypothesize the extent to which these ties ameliorate the perceived association differ across subpopulations. In general, we expect that the vulnerable and disadvantaged group benefits more from social ties. Considering older women are more resilient when facing negative life events, we expect that social ties may protect males more than females. We also expect social ties serve as more important protective barriers to the consequences of disruptive life events for rural and less educated older adults because they have less generous social security benefits and other coping repertoires.

*Hypothesis 5a: The buffering effect of social ties on negative life events is stronger for the male than the female*.
*Hypothesis 5b: The buffering effect of social ties on negative life events is stronger for older adults in rural area than those in urban area*.
*Hypothesis 5c: The buffering effect of social ties on negative life events is stronger for less educated older adults than the better-educated*.

## Data and Method

### Data

In this study, we use data from Chinese Longitudinal Aging Social Survey (CLASS) collected by the Institute of Aging Studies, Renmin University of China. The survey has been conducted for three waves in 2014, 2016, and 2018, respectively. It is a nationally representative survey of older adults in China, covering 28 provinces, 134 counties, and 462 villages/neighborhoods in China (with 34 provinces and around 2,800 counties in total). The survey uses a multi-stage stratified probability sampling method, with county as the primary sampling unit and village/neighborhood as the secondary. Within each village or neighborhood, a random sample of the households is selected, and within each household, one older adult aged 60 and over is randomly selected as the survey respondent. It collects information on their demographic and socioeconomic characteristics, access to health and services, pension/retirement planning, cognitive abilities, and attitudes toward aging.

In this study, we use the biennial panel data of 2016 and 2018 waves of CLASS, as the definition of cognitive impairment (which is used to check if the respondent is suited for answering questions regarding depressive symptoms) has been changed since 2016^1^. The 2016 wave interviewed 11,471 older adults. Among them, 9,642 respondents (84% of the total) were traced in 2018 wave, while the rest either died or lost to follow up. We only keep the participants who joined both waves and get a sample size of 19,284 person-year observations. In these two waves, each respondent needed to answer eight cognition-related questions and only those who answered five or more questions correctly would proceed to answer questions regarding depressive symptoms and attitudes. Otherwise, the respondents were regarded as cognitively impaired and not suited to answer questions regarding depressive symptoms. As a result, 8,170 person-year observations (42.37%) were excluded, and our sample reduced to 11,114 person-year observations (5,557 persons). We also drop the respondents with missing values in either wave (2,182 person-year observations, 11.3%), and most (84%) of the missing is due to missing values on our dependent variable, depressive symptom^2^. Missing data has been a common challenge in health surveys that use self-report instruments. For instance, among the 1,931 surgical patients surveyed in Canada, 351 (account for 18.2% of the total) did not fully complete the self-rated depression scale (62). The final sample size is 8,932 person-year observations (4,466 persons). We acknowledge that a missing rate of 11.3% is relatively high. But previous studies about depressive symptoms using CLASS data have shown similar (44) or even higher missing rate (63, 64), and their results were reasonable.

### Measurement

#### Depressive Symptoms

Depressive symptoms were measured by using the Center for Epidemiologic Studies Depression (CES-D) scale (65). The scale involves the frequency of 9 items of symptoms which older adults have experienced in the past week: three items measuring feelings of positive affect (feeling happy, enjoying life, feeling pleasure), two items measuring feelings of negative affect (feeling lonely, feeling upset), two items measuring feelings of marginalization (feeling useless, having nothing to do), two items measured somatic symptoms having poor appetite, having trouble sleeping. The nine-item CES-D scale has been validated and widely used in assessing Chinese older adults' mental disorder (64, 66, 67). We code the frequency with which the response had experienced each symptom in the past week as 0 (rarely or never), 1 (sometimes), 2 (often). The 9 items were summed up and a depression symptom score was created, ranging from 0 to 18, with a higher score indicating more depressive symptoms.

#### Negative Life Events

It is a binary variable and is coded as 1 if the older adults experienced any of the following 9 items of negative experiences in the previous 12 months: (1) serious illnesses; (2) natural calamities; (3) death of spouse; (4) death of child/children; (5) death of other relatives or close friends; (6) serious financial loss; (7) serious illnesses of family members; (8) experiencing problems in a relationship with family members, relatives, or close friends; (9) experiencing accidents. It's to be noted that we also used different formats of coding of negative life events (e.g., the number of negative life events, or a 3 categorical variable of negative life events), and the results are consistent (results not shown)^3^.

#### Social Ties

The Lubben Social Network Scale (LSNS) (68) is used in this paper to measure social ties for older adults. LSNS has been widely used in studies about social support for older adults in China (35, 44, 69). The scale is constructed from a set of three questions evaluating *family ties* and a comparable three questions for *friendship ties* (1) “How many relatives (including family members)/friends do you see or hear at least once a month?,” (2) “How many relatives (including family members)/friends do you feel at ease with to talk about private matters?,” and (3) “How many relatives (including family members)/friends do you feel close to such that you could call on them for help?.” Response options range from none, one, two, three to four, five to eight, nine or more. We coded each item as 0 (none), 1 (one), 2 (two), 3 (three to four), 4 (five to eight), or 5 (nine or more). The three items were summed into a scale ranging from 0 to 15 for family ties and friendship ties, separately. Then the score of social ties, the sum of family and friendship ties, ranges from 0 to 30.

#### Demographic Variables

We also control the following demographic variables in the analyses, including age, age squared, marital status (currently married or not), presence of grandchild, coresidence with adult children, self-reported health (range from 1 to 5, the higher score, the better health), number of chronic diseases (range from 0 to 24, which is measured by a list of 24 kinds of chronic conditions such as hypertension, cardiovascular diseases, diabetes, etc.) and index of functional limitations (range from 0 to 30, which is measured by how well the respondents could conduct 15 activities, such as dressing, eating, bathing, using the toilet, doing housework, etc.; the higher, the worse).

### Analytical Strategy

We use the linear fixed effects regression model to conduct analyses. Let *y*_*it*_ denotes the CES-D score for individual *i* in year *t* (i = 1,2…N; t = 1,2).


(1) $$\begin{array}{r} y_{it}=X_{it}\beta +u_{i}+\varepsilon_{it},\;i=1,2,3\ldots N;t=1,2 \end{array}$$

Where *X*_*it*_ are the time variant variables including negative life events and social ties (the social ties, family ties, and friendship ties are included in the model as continuous variables), and control variables for individual *i* in year *t*. *u*_*i*_ is the unobserved time-invariant effects which represents the individual heterogeneity, ε_*it*_ is the idiosyncratic errors which varies by individual and time.

Fixed effects model could correct for the endogeneity bias using the within transformation to eliminate time-invariant unobservable characteristics (70). For instance, in this circumstance, older adult's early life experiences might jointly influence the risk of negative life events and depression at old ages. however, fixed effects model limits our analysis only to time variant variables in the analysis. All time-invariant variables like gender and education would be removed in the demeaning process and are thus not included as control variables.

Using the fixed effects model, we first examine the relationship between negative life events and depressive symptoms controlling for all of the covariates mentioned above. We then include social ties as the key moderating variables, and differentiate the impact of family ties and friendship ties. lastly, we will also examine subsample differences by gender, rural/urban and education level attainment.

## Results

### Sample Description

Descriptive statistics of key variables for the whole sample and for three groups of subsamples are presented in Table 1. Depressive symptom as measured by CES-D score in the full sample is 6.98 on average. Female older adults suffer more severe depressive symptoms than male counterparts. There are also rural-urban and educational disparities in depressive symptoms, with rural older adults and those with less than secondary education having higher CES-D score.

**Table 1:** Descriptive statistics in total and subsamples, CLASS 2016 and 2018.

**Variable**	**Total**	**Male**	**Female**	**Rural**	**Urban**	**Less than Secondary**	**Secondary and above**
**Dependent Variable**							
CESD (range from 0 to 18)	6.98	6.93	7.04	7.20	6.80	7.11	6.80
	[2.91]	[2.87]	[2.95]	[2.78]	[2.99]	[2.82]	[3.01]
**Key independent variables**							
Negative life events	0.15	0.14	0.15	0.16	0.14	0.15	0.14
	[0.35]	[0.35]	[0.36]	[0.36]	[0.35]	[0.36]	[0.35]
Social Ties (0–30)	14.50	14.50	14.49	14.42	14.56	14.21	14.89
	[5.25]	[5.39]	[5.08]	[5.45]	[5.07]	[5.29]	[5.15]
Family ties (0–15)	7.63	7.62	7.63	7.60	7.64	7.50	7.80
	[2.69]	[2.78]	[2.60]	[2.80]	[2.60]	[2.74]	[2.62]
Friendship ties (0–15)	6.87	6.88	6.87	6.81	6.92	6.71	7.10
	[3.06]	[3.13]	[2.98]	[3.16]	[2.98]	[3.09]	[3.01]
**Covariates**							
Age	69.16	69.23	69.08	68.69	69.54	69.94	68.08
	[6.56]	[6.43]	[6.71]	[6.17]	[6.85]	[6.63]	[6.32]
Married (%)	0.77	0.86	0.67	0.77	0.77	0.73	0.83
	[0.42]	[0.35]	[0.47]	[0.42]	[0.42]	[0.45]	[0.37]
Presence of grandchild (%)	0.28	0.26	0.30	0.33	0.24	0.30	0.25
	[0.45]	[0.44]	[0.46]	[0.47]	[0.43]	[0.46]	[0.43]
Number of chronic diseases (0–24)	1.30	1.20	1.41	1.32	1.28	1.34	1.24
	[1.47]	[1.42]	[1.52]	[1.45]	[1.49]	[1.51]	[1.42]
Index of functional limitations (0–30)	0.82	0.68	0.97	0.86	0.79	0.92	0.67
	[2.31]	[2.14]	[2.49]	[2.42]	[2.23]	[2.40]	[2.18]
Self-reported health [1–5]	3.44	3.50	3.38	3.37	3.50	3.36	3.56
	[0.88]	[0.87]	[0.89]	[0.91]	[0.85]	[0.90]	[0.84]
Coresidence (%)	0.30	0.28	0.32	0.33	0.27	0.31	0.28
	[0.46]	[0.45]	[0.47]	[0.47]	[0.44]	[0.46]	[0.45]
Person-year observations	8,932	4,732	4,200	3,997	4,935	5,182	3,750

*The mean values (or percentage) are presented, with standard deviation in the parentheses*.

Overall, 14.7% of older adults have experienced at least one negative life event during the previous 12 months. Among the nine items, the three adverse life events the older adults are most likely to go through are death of other relatives or close friends, serious illnesses, and serious financial loss^4^. Female, rural, and the less educated older adults have slightly higher exposure to negative life events than male, urban, and the better-educated older adults, respectively.

The average score of social ties is 14.5, and the strength of family ties is slightly stronger than the strength of friendship ties. The score of social ties is almost identical for males and females. Older adults living in rural area and having less than secondary education have weaker family ties and friendship ties than those living in urban area and having at least secondary education.

The average age of the older adults in our sample is about 69 years old, which is almost identical among all subgroups. 77% of older adults are currently married, 29.7% of them coreside with adult children, and 27% have grandchild presented in household, with these proportions varying a little among three groups of subsamples. The female, rural, and less educated older adults have worse health with higher number of chronic diseases and functional limitations.

### Association Between Negative Life Events, Social Ties, and Depressive Symptoms

We examine the association between negative life events and depressive symptoms and address the moderating role of social ties on this perceived association. As shown in Model 1a in Table 2, after controlling for all the covariates, the CES-D score of those who have experienced at least one negative life events in the past 12 months is 0.350 higher than those who did not experience negative life events (*p* = 0.005). Moreover, the CES-D score decreases by 0.436 points with one standard deviation of increase in the score of social ties (*p* < 0.001), which means social ties are negatively associated with the severity of depression. These results are supportive of hypothesis 1 and 2.

**Table 2:** The fixed effect models predicting depressive symptoms of chinese older adults, total sample, CLASS 2016 and 2018, *N* = 8,932.

	**Model 1a**	**Model 1b**	**Model 2a**	**Model 2b**
Neg. life events (yes = 1)	0.350^***^	1.598^***^	0.348^***^	1.482^***^
	[0.126]	[0.376]	[0.126]	[0.381]
Social ties (0–30)	−0.083^***^	−0.070^***^		
	[0.009]	[0.010]		
Neg. life eventsn^*^Social ties		−0.081^***^		
		[0.023]		
Family ties (0–15)			−0.093^***^	−0.091^***^
			[0.023]	[0.025]
Friendship ties (0–15)			−0.075^***^	−0.052^**^
			[0.021]	[0.022]
Neg. life events^*^Family ties				0.028
				[0.063]
Neg. life events^*^Friendship ties				−0.186^***^
				[0.060]
Age	0.842^***^	0.814^***^	0.846^***^	0.815^***^
	[0.262]	[0.262]	[0.262]	[0.262]
Age–squared	0.001	0.001	0.001	0.001
	[0.002]	[0.002]	[0.002]	[0.002]
Married	−0.909^***^	−0.890^***^	−0.906^***^	−0.887^***^
	[0.329]	[0.329]	[0.329]	[0.329]
Presence of grandchild	−0.426^***^	−0.419^***^	−0.423^***^	−0.422^***^
	[0.141]	[0.140]	[0.141]	[0.141]
Number of chronic diseases	0.162^***^	0.154^***^	0.163^***^	0.155^***^
	[0.034]	[0.034]	[0.034]	[0.034]
Index of functional limitations	0.028	0.031	0.028	0.032
	[0.023]	[0.023]	[0.023]	[0.023]
Self-reported health (1–3, 5, 6)	−0.428^***^	−0.440^***^	−0.429^***^	−0.443^***^
	[0.059]	[0.060]	[0.060]	[0.060]
Coresidence	−0.286^**^	−0.290^**^	−0.284^**^	−0.292^**^
	[0.125]	[0.125]	[0.125]	[0.125]
Constant	−51.525^***^	−50.680^***^	−51.659^***^	−50.621^***^
	[9.151]	[9.142]	[9.157]	[9.146]
Person	4,466	4,466	4,466	4,466
*N* (Person–observation)	8,932	8,932	8,932	8,932

*Note: The coefficient values included in the models are presented, standard errors in parentheses.*

*** *p < 0.01,*

** *p < 0.05,*

* *p < 0.10 (two–tailed tests)*.

**Figure 1:**
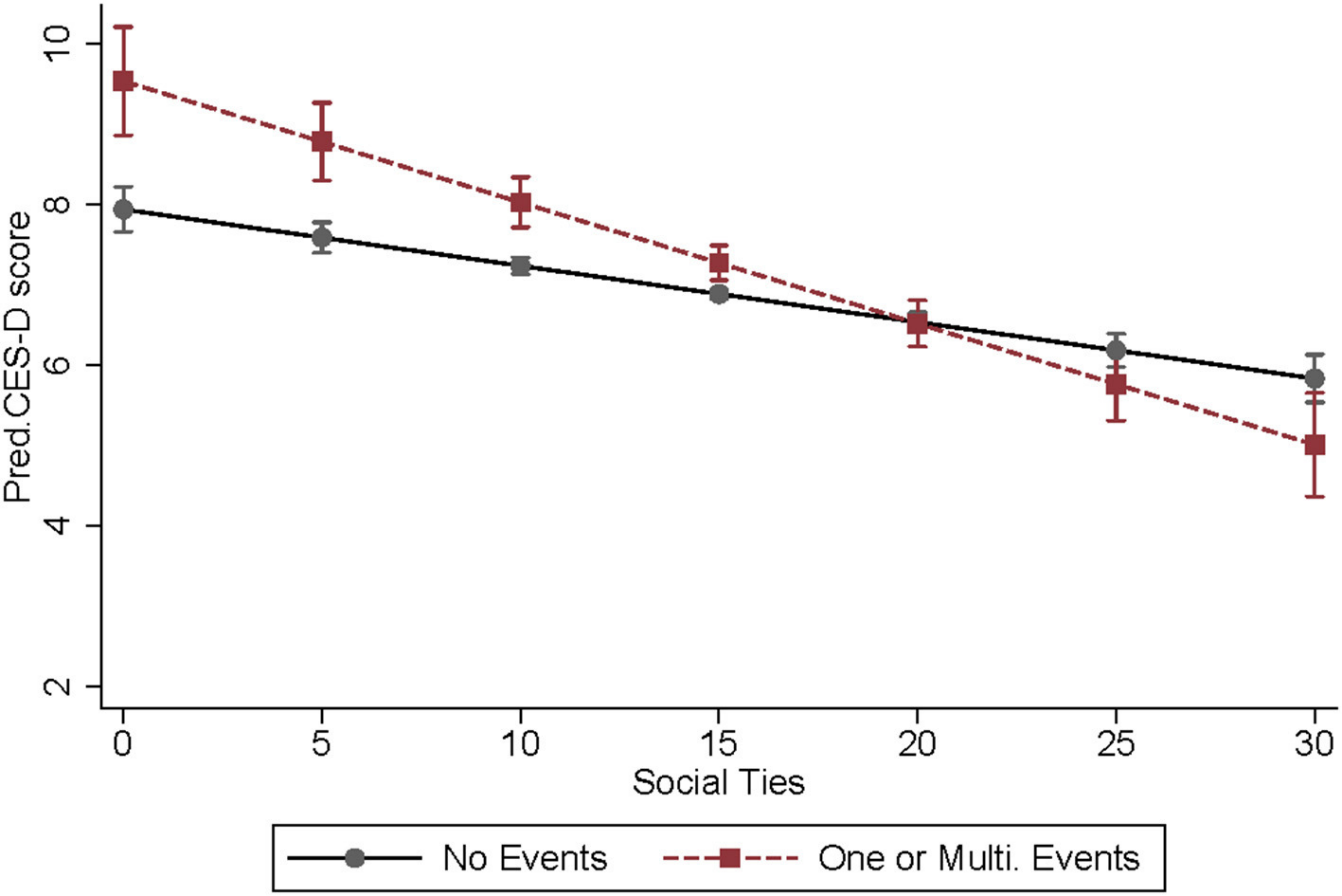
Predicted depressive symptoms by social ties and negative life events (with 95% confidence intervals), CLASS 2016–2016, *N* = 8,932.

In Model 1b, we add the interaction term of negative life events and social ties. We find that the deleterious effect of exposure to negative life events is significantly moderated by older adults' social ties (Coef. = −0.081, *p* < 0.001), which confirms hypothesis 3. To show the buffering effect of social ties more clearly, we present the predicted depressive symptoms by social ties and negative life events in Figure 1, which delivers three key information. First, the increase in the strength of social ties is associated with reduced depressive symptoms (the slope is negative either for dotted or solid lines). Second, the negative association between social ties and depression is stronger for those exposed to negative life events than for those who did not (the slope of dotted line is steeper than the slope of solid line). Third, when the social ties are weak, older adults who have experienced negative life events show more severe depressive symptoms than those who did not (a CES-D score of 9 vs. 8 when social ties score is 0). But when social ties get stronger, the gap in depressive symptoms becomes smaller, and is closed when the social ties score is around 20.

**Figure 2:**
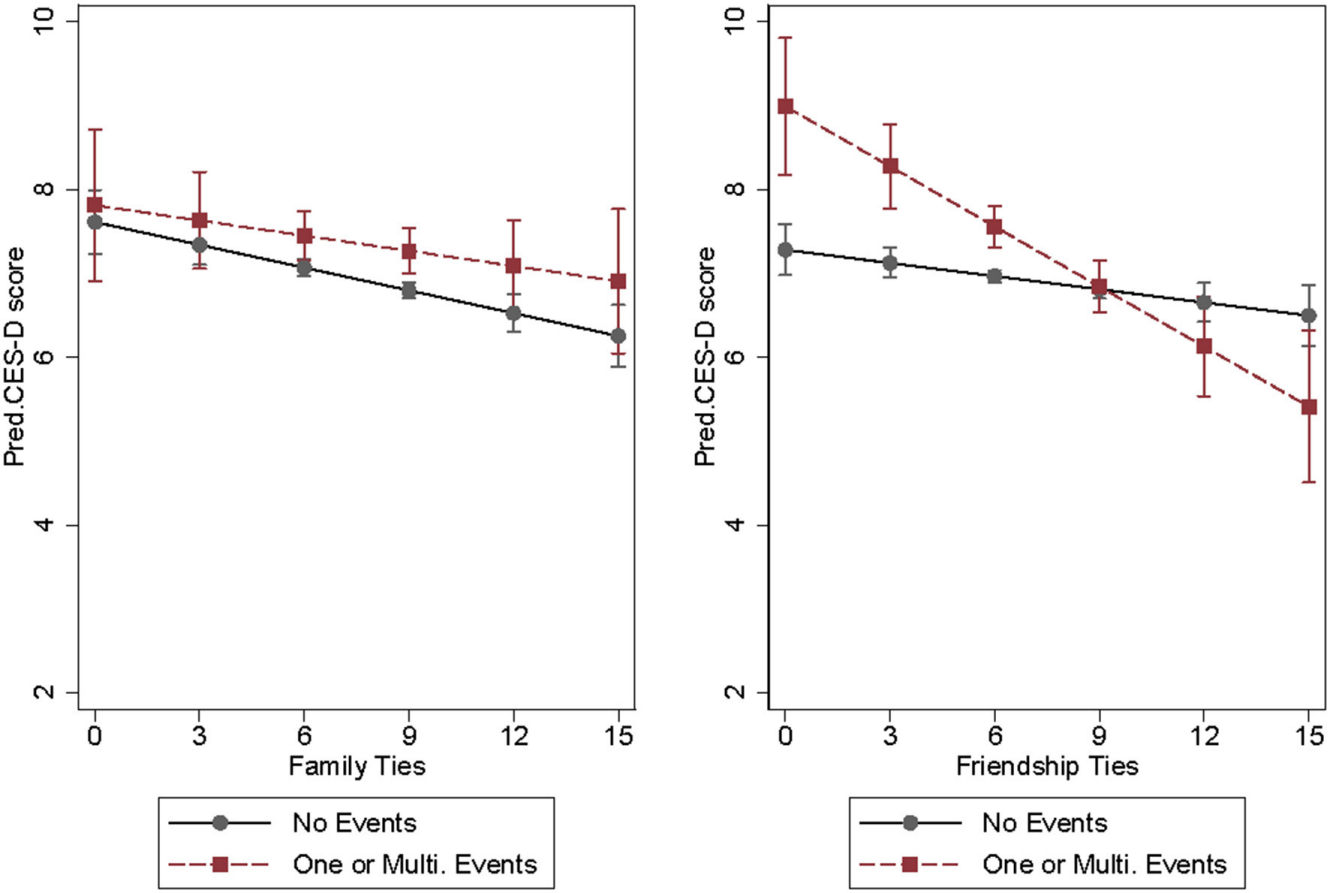
Predicted depressive symptoms by family ties, friendship ties, and negative life events, CLASS 2016–2016, *N* = 8,932.

The results from Model 1a and Model 1b indicate that social ties not only directly relate to lower depression severity, but also effectively moderate the shock from negative life events. But this does not tell us which type of social ties matters more. In model 2a and 2b, we distinguish the social ties into family ties and friendship ties to examine their differential impacts. In model 2a, both family ties and friendship ties are significantly related to lowered depression severity for older adults, and this association is stronger for family ties (coef. = −0.093, *p* < 0.001) than that of friendship ties (coef. = −0.075, *p* < 0.001). In model 2b, we further add the interaction terms of negative life events with family ties and friendship ties, separately. It turns out that only friendship ties could effectively moderate the deleterious health consequences of negative life events (coef. = −0.186, *p* < 0.001). This difference is clearly presented with the predicted depressive symptoms in Figure 2. In the left panel, regardless of the strength of family ties, the depressive symptom score for older adults exposed to negative life events is consistently higher than those who do not (the slopes of two lines are similar). But in the right panel of the graph, the gap in depressive symptoms between the groups with and without exposure to negative life events converges when friendship ties become stronger. These findings confirm hypothesis 4b which argues for the stronger moderating roles of friendship than family ties.

The covariates in the models behave in expected directions. For example, depressive symptoms increase by age. Older adults who are currently married, have less chronic diseases and functional limitations, and report better health, have a lower score of depressive symptoms. Coresidence with an adult child and presence of grandchildren can lower the depressive symptoms.

Now we move to examine whether the buffering effect of social ties (family ties and friendship ties) differs by gender and socioeconomic status of the older adults. To do so, we split our data into three groups of subsamples: male vs. female, rural vs. urban, and less than secondary education vs. secondary education and above. We focus on the interaction effects of family/friendship ties with negative life events. As is shown in Table 3, for the total sample and each subsample, the association between negative life events and depressive symptoms is significantly moderated by friendship ties, rather than family ties, which further validates hypothesis 4b. More importantly, the moderating effect of friendship ties is stronger for male, rural, and less educated older adults. In other words, the coefficient of the interaction term of negative life events and friendship ties is significantly negative for male, rural and less-educated subsamples, but remains insignificant for female, urban and better educated subsamples. This finding confirms our hypothesis 5a, 5b, and 5c, which means friendship ties play a more important role in moderating the association between disruptive life events and depression for less resilient and disadvantaged groups who have less alternative coping resources.

**Table 3:** The fixed effects models predicting depressive symptoms of Chinese older adults, by subsamples, CLASS 2016 and 2018, *N* = 8,932.

	**Male**	**Female**	**Rural**	**Urban**	**Less than secondary**	**Secondary and above**
Neg. life events	1.426^***^	1.559^***^	1.791^***^	0.312	1.666^***^	1.251^*^
	[0.503]	[0.588]	[0.541]	[0.578]	[0.472]	[0.641]
Family ties (0–15)	−0.073^**^	−0.114^***^	−0.117^***^	−0.098^***^	−0.064^**^	−0.141^***^
	[0.034]	[0.038]	[0.037]	[0.037]	[0.032]	[0.040]
Friendship ties (0–15)	−0.035	−0.067^**^	−0.05	−0.066^**^	−0.086^***^	−0.007
	[0.030]	[0.033]	[0.032]	[0.033]	[0.028]	[0.035]
Neg. life events*Family ties	0.136	−0.093	−0.003	0.072	0.015	0.059
	[0.084]	[0.095]	[0.092]	[0.092]	[0.077]	[0.107]
Neg. life events^*^Friendship ties	−0.310^***^	−0.046	−0.209^**^	−0.087	−0.230^***^	−0.147
	[0.083]	[0.089]	[0.086]	[0.093]	[0.073]	[0.105]
Age	0.457	1.171^***^	0.196	1.385^***^	0.309	1.209^***^
	[0.366]	[0.377]	[0.390]	[0.357]	[0.329]	[0.440]
Age–squared	0.003	−0.001	0.004	−0.002	0.004^*^	−0.001
	[0.003]	[0.003]	[0.003]	[0.003]	[0.002]	[0.003]
Married	−1.224^***^	−0.521	−0.661	−1.476^**^	−0.840^**^	−1.009
	[0.458]	[0.476]	[0.418]	[0.616]	[0.367]	[0.683]
Presence of grandchild	−0.451^**^	−0.395^*^	−0.412^**^	−0.441^**^	−0.377^**^	−0.427^*^
	[0.192]	[0.207]	[0.194]	[0.218]	[0.176]	[0.230]
Number of chronic diseases	0.120^**^	0.189^***^	0.161^***^	0.097^**^	0.189^***^	0.117^**^
	[0.047]	[0.050]	[0.056]	[0.046]	[0.044]	[0.054]
Index of functional limitations	0.031	0.035	0.054^*^	0.006	0.021	0.045
	[0.031]	[0.033]	[0.033]	[0.032]	[0.028]	[0.039]
Self–reported health (1–3, 5, 6)	−0.514^***^	−0.378^***^	−0.157^*^	−0.730^***^	−0.461^***^	−0.419^***^
	[0.081]	[0.088]	[0.081]	[0.090]	[0.074]	[0.099]
Coresidence	−0.317^*^	−0.276	−0.03	−0.414^**^	−0.327^**^	−0.222
	[0.174]	[0.180]	[0.174]	[0.189]	[0.157]	[0.204]
Constant	−37.151^***^	−64.072^***^	−23.362^*^	−73.676^***^	−30.792^***^	−66.915^***^
	[12.752]	[13.159]	[13.517]	[12.521]	[11.597]	[15.111]
Person	2,366	2,100	2,143	2,612	2,591	1,875
*N* (Person–observation)	4,732	4,200	3,997	4,935	5,182	3,750

*The coefficient values are presented, standard errors in parentheses.*

*** *p < 0.01,*

** *p < 0.05,*

* *p < 0.10 (two-tailed tests)*.

## Discussion and Conclusion

Based on the nationally representative elderly survey in China, this paper examines the association between negative life events and depressive symptoms for older adults and explores how the perceived association is contingent on the strength of social ties. Our paper confirms that exposure to negative life events is an important risk factor of depression for older adults, echoing the previous findings for older adults in Singapore and Hong Kong of China (14, 16). Moreover, older adults with stronger social ties display less severe depressive symptoms than those with weaker social ties when experiencing negative life events. This finding in the context of mainland China enrich the evidence on the moderating roles of social ties, in support of the social convoy model ((38), p. 84).

More importantly, our paper expands on the current literature to compare the relative importance of family ties and friendship ties. On the one hand, both family ties and friendship ties are directly related to less severe depressive symptoms. On the other hand, the association between negative life events and depression is significantly moderated by friendship ties rather than family ties. Also, the moderating roles of friendship ties is more prominent for male, rural and less educated older adults, who are less resilient and have limited access to alternative coping resources. There might be two possible explanations for the stronger moderating roles of friendship ties as compared with family ties. First, the patriarchal culture strengthens family ties and intergenerational solidarity, but at the same time, it emphasizes the primary power and authority of male seniors and hinders intimate emotional interaction within household. Older adults might not be adapted to sharing painful and embarrassed feelings in case of stressful events with close family members (71, 72), thus friendship ties turn to be more important to cushion the shock. Second, the most common disruptive life event at old ages is death of family members. Family thus may become a pool where negative emotions and conflicts accumulate (73, 74), leading to a reduced buffering effect of family ties.

This paper also has several limitations. First, we code the exposure to negative life events as a dummy variable. Future studies could further differentiate the types of disruptive life events, such as family bereavement, severe illness, divorce, economic strains, and others. As such, they can figure out how social ties function to withstand the shock of various kinds of negative life events. Second, for each wave of the CLASS survey, self-reported negative life events are measured cross-sectionally and might be subject to recall bias. For instance, older adults with depressive symptoms tend to perceive usual experience more negatively and report it as a negative life event, leading to an overestimation of the association between negative life events and depression. In order to evaluate the severity of such bias, we conduct a sensitivity test by restricting the scope of negative life events to these less prone to recall bias such as the death of spouse/children/relatives, natural calamities, and accidents. The results remain robust, but we still need to be mindful of potential bias^5^. Third, although we use fixed effects model to correct for the endogeneity bias resulting from time-invariant omitted variables, the endogeneity bias resulting from reversed causality might persist. For instance, severe depressive symptoms might lead to weakening friendship ties, such as, isolation from their friends, while have lesser impacts on family ties due to mutual obligations and commitments. Therefore, we should be cautious to attach a causal interpretation to our regression results. Future studies should use more rigorous design and longitudinal data to verify the time-varying effects of social ties on the association between negative life events and depression among the elderly.

Despite these limitations, our findings offer fresh evidence on the different roles of family and friendship support in moderating the association between stressful life events and depression in the context of mainland China, the society faced with accelerated aging process and intensified health concern. With heightened risk of encountering disruptive life events at old ages, the older adults are more susceptible to depression, which has become a major health challenge in China. Current policies often highlight the consolidation of family network to improve the well-being of older adults. For instance, the Central Committee of the Communist Party of China and the State Council jointly released a guideline addressing the issue of population aging in November 2021, that explicitly encourages adult children to co-reside or live^5^ close to their elderly parents. However, our study suggests a new and complementary perspective that highlights the importance of friendship ties for older adults' psychological health. Social ties outside the family, such as contact with and counseling from close friends, could act as a pivotal coping resource in relieving stresses from disruptive life events for older adults, in particular for those who are less resilient and have less access to alternative coping resources. Therefore, building and strengthening social network is as important as family network in promoting healthy aging in China.

## Data Availability Statement

The raw data supporting the conclusions of this article will be made available by the authors, without undue reservation.

## Ethics Statement

Ethical review and approval was not required for the study on human participants in accordance with the local legislation and institutional requirements. Written informed consent for participation was not required for this study in accordance with the national legislation and the institutional requirements.

## Author Contributions

HR and FC analyzed data and interpreted the results. HR and KS drafted and revised the paper. All authors have read and approved the final version of the manuscript.

## Funding

This work was supported by National Social Science Foundation of China (21&ZD189) and National Science Foundation of China (72073032).

## Conflict of Interest

The authors declare that the research was conducted in the absence of any commercial or financial relationships that could be construed as a potential conflict of interest.

## Publisher's Note

All claims expressed in this article are solely those of the authors and do not necessarily represent those of their affiliated organizations, or those of the publisher, the editors and the reviewers. Any product that may be evaluated in this article, or claim that may be made by its manufacturer, is not guaranteed or endorsed by the publisher.

## Footnotes

^1^In 2014 wave of CLASS, they use five cognition-related questions and only those answered at least three questions correctly were invited to finish the mental health and attitudes questions, but since 2016 three more questions were added and those who answered at least 5 questions correctly were able to participate in the mental health and attitudes questions.

^2^1106 person-year observations were missing in the CES-D, and another 728 person-year observations were dropped in Fixed-effects model because only one wave of the data has record in CES-D.

^3^For example, we use the number of negative life events (range from 0 to 4) as the key independent variable and the result shows that one more negative life events will lead to 0.265 points of increase in CES-D score (*p* = 0.010); compared with those did not experience negative life events, those who experience one event will have 0.338 points higher CES-D score, and those who experienced multiple (≥2) events will be 0.419 points higher. But considering that the sample size of those who experienced multiple events is quite small (only about 1.87% of the sample), we use the binary negative life events in our following analysis.

^4^Due to space limitation, we do not present the detailed summary statistics of these nine negative life events.

^5^Due to space limitation, the results are not shown in the paper and are available upon request.
